# Is Papaya Leaf Extract the Sweet Remedy for Dengue-Induced Thrombocytopenia?

**DOI:** 10.7759/cureus.71852

**Published:** 2024-10-19

**Authors:** Sheneel Jaggernauth, Shyam R Ramoutar, Renuka Ramoutar, Neiala Piaralal

**Affiliations:** 1 Department of Medicine, Eric Williams Medical Sciences Complex, Champ Fleurs, TTO; 2 Department of Opthalmology, Eric Williams Medical Sciences Complex, Champ Fleurs, TTO; 3 Department of Medicine, Medical Associates Hospital, Champ Fleurs, TTO

**Keywords:** conventional care, dengue, medicinal properties, papaya leaves, platelet count, rna virus

## Abstract

Dengue fever, caused by the dengue virus and transmitted by *Aedes mosquitoes*, poses a significant health challenge in tropical regions like Trinidad and Tobago, with severe cases leading to life-threatening complications. This case study presents a 65-year-old female with dengue exhibiting warning signs, characterized by persistent fever, abdominal pain, and thrombocytopenia. Despite supportive care, including intravenous fluids and symptomatic treatment, her platelet count remained critically low at 12.1 x 10^9^/L. Following the introduction of diluted papaya leaf extract into her treatment regimen, her platelet count significantly improved to 122.7 x 10^9^/L within two days, eventually returning to normal at follow-up appointments. This case suggests that Carica papaya leaf extract may provide therapeutic benefits in boosting platelet counts in dengue patients when used alongside conventional supportive care. Further research is essential to understand the extent of papaya’s benefits and to integrate it safely into dengue management practices, potentially enhancing treatment options for affected individuals. In addition, public health efforts should be prioritized in raising awareness about dengue prevention, early diagnosis, and effective management strategies to reduce the disease's impact in affected regions.

## Introduction

Dengue virus (DENV) is an arboviral illness belonging to the Flaviviridae family and has four serotypes (DENV1, DENV2, DENV3, DENV4), which are commonly found in tropical and subtropical regions worldwide, particularly endemic in the Caribbean, such as Trinidad and Tobago. Each year, up to 400 million people are affected by dengue [1]. The main route of transmission is through the bite of a female *Aedes aegypti* mosquito. Most patients are asymptomatic, but some may develop symptoms that can be life-threatening, with a case fatality rate of less than 1% in Trinidad and Tobago [2]. 

Dengue can present with or without warning signs. Common symptoms include fever, headaches, retro-orbital pain, severe arthralgia and myalgia, and a maculopapular rash. Patients exhibiting warning signs may experience abdominal pain, persistent vomiting, lethargy, an enlarged liver, fluid leaking from the intravascular region, and hemorrhagic manifestations such as petechiae or epistaxis. Severe dengue, also known as dengue hemorrhagic fever, can further deteriorate into dengue shock syndrome [1]. Severe dengue is the leading cause of death related to dengue infection, caused by severe hemorrhage, plasma leakage, fluid accumulation, respiratory distress, or organ impairment. Previous studies showed a significant number of DENV‐2 infected patients developed severe dengue more frequently as compared to other serotypes [3]. 

Treatment focuses on supportive care, as there is no specific antiviral therapy. Some anecdotal evidence suggests that papaya leaves may help increase platelet counts, potentially aiding recovery by boosting the immune response. While it is crucial to consult healthcare professionals for proper management, exploring natural remedies like papaya leaves could complement already established therapies [4].

This case presentation highlights a 65-year-old female diagnosed with DENV exhibiting warning signs. It explores the management protocols implemented to ensure an optimal prognosis and quality of life.

## Case presentation

A 65-year-old female with no known medical conditions who lives near an area of dense vegetation presented to the hospital on September 4, 2024, with a two-day history of fever and body pains. The patient reported several episodes of vomitus, which had food contents, no blood or bile, and was unable to quantify the amount. The fever was objective (39°C) and continuous, with relief from paracetamol but none from tepid sponging, and associated with no chills and severe generalized body pains. The patient also complained of abdominal pain, which was rated at a severity of 2/10, dull in nature with no radiation and no transformative factors. She denied any bleeding, headaches, retro-orbital pain, rashes, myalgia, or arthralgia and the review of systems were unremarkable. 

On clinical examination, the patient was lying comfortably with no signs of obvious cardiopulmonary distress, oriented to time, place and person with a Glasgow Coma Scale (GCS) of 15/15. She had a blood pressure of 115/70 mmHg, a pulse of 73 bpm, a respiratory rate of 20 breaths per minute, SpO_2_ of 95% on room air, and a temperature of 39°C. On palpation, the abdomen was soft and nontender whilst the liver was firm and enlarged 2 cm below the right costal margin. On auscultation, the patient had normal bowel sounds. Examination of the respiratory, cardiovascular and neurological systems were unremarkable. 

Investigations

This patient’s initial workup on the 4/09/2024 included random blood sugar (RBS), urinalysis, blood investigations, NS1 antigen, COVID-19 antigen, electrocardiogram (ECG), and chest X-ray (CXR). This patient’s RBS was 114 mg/dl, urinalysis showed no positive finding, COVID-19 antigen negative, and HIV negative, while the NS1 antigen test and IgM was positive. CXR showed no remarkable findings other than cardiomegaly. A 12-lead ECG showed a rate of 66 bpm and sinus rhythm without ischemic changes. No echocardiogram was done as it was not available. Complete blood count (CBC) and liver function tests (LFTs) trends were conducted, and the results are also included in Table 1. In addition, an initial set of tests, including renal function tests (RFTs), lipid profile, HbA1c, and blood group, and crossmatch, were performed for this patient, with results tabulated below in Table 2.

**Table 1:** Blood investigation trend

Laboratory investigation	Reference value	04/09/24	5/09/24	6/09/2024	7/09/24
Hemoglobin (g/L)	12-16	12.78	13.4	13.5	13.4
Mean corpuscular volume (fL)	80-100	87.20	94.3	95.1	93.6
Hematocrit (%)	40-50	43.2	41.4	45.6	44.3
Leukocyte (*10^9/L)	4.5-11	7.04	7.01	8.91	7.71
Thrombocyte (*10^9/L)	150-400	13.30	12.10	122.1	160.7
Albumin (g/dL)	3.5-5.5	3.0	4.1	4.2	4.1
Total bilirubin (mg/dL)	0.3-1	0.6	1.2	1.0	0.9
Unconjugated (mg/dL)	0.2-0.8	0.4	0.9	0.6	0.6
Conjugated (mg/dL)	0.0-0.3	0.2	0.3	0.4	0.3
Alkaline phosphatase (IU/L)	40-129	54	110.6	107.4	104.7
Gamma-glutamyltransferase (IU/L)	8-61	43	32	28	30
Aspartate transferase (U/L)	8-48	298	243	124	96
Alanine transaminase (U/L)	7-55	45	51.2	40.6	42.2

**Table 2:** RFTs, lipid profile, and HbA1C (%) done on 4/9/2024 RFTs: renal function tests, HbA1C: hemoglobin A1C

Laboratory investigation	Reference value	4/09/24
Serum creatinine (mg/dL)	0.6-1.2	0.58
Blood urea nitrogen (mg/dL)	6.0-23	8.0
Sodium (mmol/L)	136-145	131
Potassium (mmol/L)	3.5-5.1	3.7
Chloride (mmol/L)	98-197	98.0
Cholesterol (mg/dL)	50-200	170
Triglycerides (mg/dL)	<150.00	103.3
High-density lipoprotein (mg/dL)	35-55	44
Low-density lipoprotein (mg/dL)	<100.00	91.0
HBA1C (%)	4.8-5.9	4.4

The patient's treatment included supportive care with intravenous fluids at a rate of 100 mL/hr, paracetamol 1 g intravenously every eight hours, dimenhydrinate 50 mg intravenously every eight hours or as needed, and esomeprazole 40 mg orally once daily. She was advised to maintain strict bed rest and follow a normal diet. A mosquito net was provided, and the patient was closely monitored for any signs of bleeding during her four-day stay in the ward. The patient's HCT levels remained stable and within normal limits, suggesting adequate fluid management and no significant bleeding complications, which is particularly important in the context of thrombocytopenia often seen in dengue patients. On September 5, 2024, a repeat CBC showed persistent thrombocytopenia with a platelet count of 12.1 x 109/L. The patient's family introduced diluted papaya leaf extract (25 mL of water), which she consumed twice daily. By September 6, 2024, her platelet count began to rise, reaching 122.7 x 109/L, and continued to improve on September 7, 2024, as shown in Table 1. The initial elevated AST indicates potential liver involvement typical in dengue infections. The subsequent normalization of AST levels reflects the improvement in liver function as the patient's clinical status improved, underscoring the importance of monitoring liver enzymes in such cases. Upon discharge, the patient was counseled on the importance of adequate fluid intake, as well as the use of antipyretics and antiemetics. She continued to drink the papaya leaf extract for an additional two days, totaling five days of consumption. Follow-up appointments were scheduled for the day after completing the papaya juice extract and one week later. Repeat CBCs conducted at both follow-ups showed that her platelet count was within the normal range (162 x 10^9^/L and 164 x 10^9^/L) , and she remained asymptomatic. Her hematocrit count was stable at 44% as well. Overall, the patient showed significant improvement in her condition, with effective monitoring and supportive care contributing to her recovery.

## Discussion

Dengue fever is a significant global health concern, particularly in tropical and subtropical regions [1]. One of the most critical complications of dengue is thrombocytopenia, characterized by a decrease in platelet count, which can lead to severe bleeding and increased morbidity and mortality. Thrombocytopenia in dengue is thought to result from multiple factors, including bone marrow suppression, increased platelet destruction due to viral infection, and the immune response. In cases of dengue, thrombocytopenia is often most pronounced during the critical phase of the illness, which typically occurs around days three to seven of the illness. Overall, thrombocytopenia is a critical factor in assessing the prognosis and management of dengue fever.

Dengue can be managed with supportive care, including intravenous fluids for proper hydration, pain and nausea relief, and careful monitoring for warning signs. A live attenuated vaccine is available in dengue-endemic regions for individuals aged nine to 45 or nine to 60 who have tested positive for dengue and are neither immunocompromised nor pregnant; however, this vaccine is not available in Trinidad and Tobago [4]. Public health efforts should prioritize raising awareness and educating communities about dengue prevention, early diagnosis, effective management, and environmental control strategies to reduce the disease's impact. While efforts to develop specific antiviral treatments have faced challenges due to the virus's mutations and serotypes, alternative therapies aimed at improving platelet counts, such as *Carica papaya* extract, have garnered interest as potential adjunctive treatments [5].

*Carica papaya*, commonly known as papaya, contains various phytochemicals, including flavonoids, vitamins, and enzymes, which may contribute to its therapeutic effects. Papaya is known for its antioxidant, anti-thrombocytopenic, and anti-inflammatory properties [6]. A study indicates that the active components of papaya enhance the expression of the arachidonate 12-lipoxygenase (ALOX 12) and platelet-activating factor receptor (PTAFR) genes, resulting in a rise in megakaryocyte production and their transformation into platelets [7-8]. Flavonoids in papaya leaf extracts contribute to their ability to stabilize membranes, potentially preventing internal bleeding in blood vessels, enhancing overall immune response [6,9]. Notably, the flavonoid quercetin can inhibit DENV replication by targeting the NS2B/NS3 protease, crucial for processing structural proteins in new virions. Therefore, compounds that inhibit this protease are promising candidates for antiviral therapies [10].

Case reports indicate significant increases in platelet levels among patients consuming papaya leaf extract, especially during critical phases of dengue when platelet counts are at their lowest. However, controlled clinical studies have yielded mixed results; some randomized trials show statistically significant improvements in platelet counts among those treated with *Carica papaya* extract, while others report no significant differences. This variability highlights the need for further research to establish the extract's effectiveness [11].

In this study, the patient received 25 ml of *Carica papaya *extract for five days, administered twice daily, alongside fluid hydration and symptomatic treatment. *Carica papaya* extract generally appears safe when consumed in moderation; however, its efficacy may vary based on individual factors such as disease severity, genetic predispositions, and overall health status [11]. In addition, some studies suggest that the dose and treatment duration may not significantly impact its effectiveness [11].

It is also important to consider the broader public health initiatives that could mitigate the impact of this disease. First, vector control remains a cornerstone of prevention where environmental management measures such as eliminating standing water and regular insecticide spraying are crucial. Community clean-up campaigns can also significantly reduce mosquito breeding sites. Educating the community about dengue transmission, symptoms, and preventive measures like using repellents and wearing protective clothing can empower individuals to protect themselves. In addition, implementing effective surveillance systems is vital. Tracking dengue cases and monitoring mosquito populations will allow for timely interventions, especially during outbreaks. Engagement with local communities is essential. Training community health workers to lead prevention efforts can foster a sense of ownership and responsibility within the community.By addressing these public health initiatives alongside individual cases, we can better understand how to manage and ultimately reduce the incidence and severity of dengue in our communities [2,12].

​​While preliminary evidence suggests that *Carica papaya *extract may help increase platelet counts in patients with DENV infection, further well-designed, large-scale clinical trials are necessary to establish its efficacy and safety. Understanding the precise mechanisms by which papaya affects platelet production could lead to more effective interventions and potentially enhance existing treatment protocols for dengue fever. Although it shows promise as a supportive treatment, it should be considered a complementary approach rather than a substitute for conventional medical care, ensuring comprehensive management of dengue patients. 

## Conclusions

The management of dengue fever remains challenging due to the absence of specific antiviral therapies and the risks associated with severe complications such as thrombocytopenia. This case shows that *Carica papaya *leaf extract may provide therapeutic benefits, by boosting platelet counts in dengue patients just after using it for five days, twice a day when used in conjunction with supportive care in Trinidad and Tobago. While supportive care is essential, increasing awareness of severe disease associated with warning signs and enforcing public health initiatives is vital for improving patient outcomes. Further research with controlled studies is needed to investigate the potential therapeutic effects, efficacy, and safety of *Carica papaya* in relation to dengue infection. By deepening our understanding of the medicinal properties of *Carica papaya*, we can improve treatment options for those affected by dengue.
